# Social purpose in an organization from the perspective of an employee: a self-determination outlook on the meaning of work

**DOI:** 10.1186/s13104-020-05432-4

**Published:** 2021-01-07

**Authors:** Malwina Puchalska-Kamińska, Agnieszka Łądka-Barańska, Marta Roczniewska

**Affiliations:** 1 Institute of Psychology, SWPS University of Social Sciences and Humanities, Warsaw, Poland; 2 Institute of Psychology, University of Gdansk, Gdansk, Poland; 3 Procome Research Group, Medical Management Center, Department of Learning, Informatics, Management and Ethics, Karolinska Institutet, Stockholm, Sweden; 4 Center of Research On Cognition and Behaviour, Institute of Psychology, Faculty in Sopot, SWPS University of Social Sciences and Humanities, Sopot, Poland

**Keywords:** Corporate social responsibility, Meaning of work, Self-determination theory, Autonomy, Competence, Relatedness, Case study, Employee volunteering

## Abstract

**Objective:**

Advancing social purpose in organizations is usually studied from the macro perspective, i.e., how it benefits organizational business goals or society more broadly. In this paper, we focus on social purpose from the perspective of the employee and propose that advancing social purpose in an organization allows individuals to fulfil an important human need for the meaning of work (MW). This study’s objective was to assess whether a volunteering Corporate Social Responsibility (CSR) program in a manufacturing company allows employees to fulfil their basic psychological needs for relatedness, competence, and autonomy. The data was collected through in-depth interviews with 15 employees and an analysis of artifacts.

**Results:**

In the analysis, three main themes describing different aspects of voluntary work at the company were identified. We found that across all groups of interviewed employees the voluntary activities served the needs of (1) relatedness, (2) competence, and (3) autonomy. We conclude that CSR programs have the most positive impact on MW when they allow employees to engage in prosocial actions and satisfy those needs.

## Introduction

Recent research demonstrates that finding the meaning of work (MW) is a growing need among employees [1]. MW is the subjective experience of significance and intrinsic value in one’s work [2]. Definitions of MW highlight an individual’s need to make sense of one’s self [3], find a sense of purpose in work [4, 5], and the desire to serve the greater good [6]. Based on Bakan’s [7] framework of two fundamental modalities of human existence (agency and communion), two aspects of MW can be derived [8]. *Agentic MW* relates to perceiving one’s work as meaningful to the extent to which the work brings personal benefits: enhances the meaning of one’s life, contributes to a sense of self-development, and allows to accomplish goals central to the self [8]. *Communal MW* refers to the degree to which an employee perceives the work as having a beneficial impact on other people or ‘world’ in general: it involves viewing one’s work as a calling, a sense of fulfilling a mission at work, and acting for the good of the humanity or the environment [8].

Corporate social responsibility (CSR) programs, designed to serve a greater good, seem to have a potential to support the need for MW among employees, especially in its communal aspect. The problem is that many of the CSR initiatives are ill-suited to fulfil this need because they are often planned top-down, precluding employees from directly engaging in prosocial activities. This ‘distance’ can impede creation of communal MW for individual employees. In fact, research shows that most employees have little knowledge about their firm’s CSR activities, and increasing employees’ proximity to the CSR initiatives is seen as a major challenge for managers [9]. Yet, only active involvement in CSR activities relates to the meaning of work [10]. Following self-determination theory (SDT) [11, 12], we argue that prosocial actions in an organization create individual MW when they involve intrinsic (i.e., inherent drive) rather than extrinsic (i.e., external regulation) motivation.

The basic psychological needs for relatedness, competence, and autonomy are the basis of intrinsic motivation and behavior [13]. *Relatedness* reflects the extent to which a person feels that he or she “belongs”, i.e., is connected to other people. This need is fulfilled when individuals are able to interact with others, experience caring for them [14], and have a sense of relevance in their lives [15]. CSR activities that are focused on helping others are inherently interpersonal. Relatedness is also developed by setting common goals, which builds a sense of cohesiveness in a team. *Competence* represents a sense of efficacy: the notion of being able to achieve one’s goals [13]. To satisfy this need one ought to feel effective, i.e., perform a behavior that has a positive effect on the world [13]. Competence is related to the sense of mastery: an experience of being good at what one does [13]. *Autonomy* signifies undertaking decisions and actions with a sense of volition and internal locus of control. An autonomous act is considered to be an expression of one’s values and reflects the self. To fulfil the need for autonomy, the actions need to be experienced as self-initiated, self-chosen, or self-endorsed. Thus, extrinsic awards or others-imposed conditions hamper intrinsic motivation [16].

Fulfilment of relatedness, competence, and autonomy needs is linked with positive employee outcomes [17]. Additionally, enhancement in well-being from prosocial behavior is mediated by the satisfaction of autonomy, competence and relatedness [18]. It follows, therefore, that CSR activities may be instrumental in creating communal MW as long as they serve the basic needs enlisted by SDT. In this article, we study a case of a manufacturing company whose business goals are not devoted to social purpose, like teaching, curing or helping. Thus, communal MW cannot be simply achieved by the employees by means of pursuing a job with a clear calling. Here, we focus on one of the company’s core CSR activities, i.e., volunteer work, to answer a question whether it allows employees to fulfil the basic needs outlined by SDT.

## Main text

### Materials and methods

#### Organizational context

The study was conducted in a manufacturing company in Poland, which currently employs over 80 people (c. 50% white collar employees). Voluntary work in this company is a part of their CSR. Each employee engages a minimum of 16 working hours in voluntary actions per year. There is a list of voluntary projects from which employees can choose activities, and they can submit new projects. Additionally, 1.5% of each employee's remuneration is allocated to the charity budget.

#### Data collection and methods

The study used qualitative research method. The data was collected through in-depth interviews and an analysis of artifacts, i.e., multimedia materials like employer branding ads and lectures where managers share knowledge about leadership. Semi-structured interviews with 15 employees from different departments were conducted: five blue-collar employees, five white-collar employees, and five managers. The interview guide contained questions about the characteristics of work, employee attitudes, and voluntary activities of employees (see Additional file 1). The interviews lasted 45 to 90 min, were recorded, and transcribed verbatim.

Based upon SDT, three categories of codes were derived: relatedness, competence, and autonomy. Two team members (MPK, AŁB) independently reviewed the interviews and assigned the codes. Discrepancies were discussed and final decisions made by the whole team.

### Results

In the analysis, three main themes related to SDT theory were derived. They described how voluntary activities served the needs of relatedness, competence, and autonomy. The themes are illustrated with quotes that are poignant and representative of the findings (see Table 1). The results are discussed for the three groups jointly (i.e., managers [M1–M6], white collar employees [WC1–WC6], blue collar employees [BC1–BC6]), but we also point to differences in their experiences and perceptions.

**Table 1:** Quotations from three groups of research participants divided into three identified themes

Position	Relatedness	Competence	Autonomy
Managers (M)	M1. *"After a year, you meet the person you helped, and they remember you. It is a pleasant feeling."*	M3*. "You have to try new things and leave your comfort zone while helping."*	M5. *"I can submit any project for consideration. I do this through an internal tool where I describe the situation and what sort of help is needed. Then all employees can vote to make it happen.”*
	M2*. "The Foundation is not separated from the company and its employees, at least we don't see it that way. The foundation is 'us' and we are the foundation."*	M4*. “You can provide long-term support and change people’s lives in a positive way.”*	M6. *"We treat ourselves as adults. If we commit to volunteering, the company trusts that we will fulfill it."*
White-collar (WC)	WC1. *"We invite our families and friends to join these actions* *It is a positive experience for everyone—spending time together and doing something valuable. It integrates us.”*	WC3*. "While interviewing family, we learn to have difficult conversations—to ask difficult, uncomfortable questions and to accept that someone may not want to answer."*	WC5. *"We all decide where our money from the charity fund goes."*
	WC2. *“We have to face difficult situations while helping, for example, during a difficult conversation with recipients of our help, to whom we have to set psychological boundaries. We can rely on ourselves in these situations. We know that we are together, and this builds trust.”*	WC4. *"We can organize help to families and friends in need. Thanks to this we know that we can help when the need arises. I know that I can help directly. I don't have to send them anywhere else.”*	WC6. *"I like planting trees and spending time outdoors. I choose such voluntary actions that fit my preferences."*
Blue-collar (BC)	*BC1. "My wife works in a prosocial organization, and we are co-organizing a fair with this place. It is a win–win strategy—children get money, and we are happy that we can help."*	BC3*. "I volunteer when I feel competent in a certain area. If I feel good at something, I will gladly use this skill. I used to work in a renovation company so I took part in a project to renovate the apartment of a lady living off a pension with a family of many children."*	BC5. *"I do not have too many of my own charity initiatives. I am happy to contribute to someone else's initiative."*
	BC2. “*We all work together. We know who has what skills and we help each other and trust each other, like in a family.”*	BC4. *"It brings such a joy when you see that you have helped someone. Someone who really needed it."*	BC6. *“Nobody is forcing me to do anything. Lately, I have little time for after-work activities—small children—so I'm less involved.”*

*M* Managers, *WC* White-collar employees, *BC* Blue-collar employees

#### Relatedness

One of the most distinctive characteristics of this volunteering program is the focus on local environment and personal contact. An example of this is an annual action of preparing Christmas gifts for the families in need who come from the city where the company is based. All employees are involved in this project. They form teams representing different departments and are responsible for finding a family that needs help. The gifts are delivered in person. These activities allow employees to create bonds with the help recipients (M1). As employees often invite their families to help, they feel that they are building a larger community of people who care for others. Spending time together with colleagues’ relatives is considered a bonding experience (WC1, BC1).

Although projects are conducted in small teams, employees celebrate and exchange their experiences at the organizational level, e.g., during Christmas party, employees describe their Christmas gift project and show pictures/videos of the experience. Respondents expressed that helping under the auspices of their organization allows them to create a sense of mutual goals and values with other team members. Some of them declared that volunteering and dealing with potentially difficult situations together builds trust towards their coworkers (WC2). They often used metaphors of “a family” or “a sports team” when describing their organizational community (M2, BC2). They viewed the work at the company and their charity work as coherent, and they identified with both of them.

#### Competence

The fact that employees can freely choose a voluntary activity rather than being assigned to one, gives them an opportunity to utilize their strengths. Blue-collar workers focused mostly on being able to utilize their sills—they declared that they help in a way that is possible for them and that involves their skills and knowledge (BC3). Thus, they perceived their input from the perspective of being ‘useful’. Managers and white-collar workers, one the other hand, saw the wide variety of possible voluntary activities as a chance to develop their competences or learn new things (M3, WC3). For example, helping others in need allowed them to develop necessary skills to deal with difficult emotions or situations.

As the projects are focused on solving concrete problems and are well-planed, respondents deemed observing the short- and long-term effects of their efforts as rewarding (M4). Being personally engaged in helping enables them to perceive the positive consequences of their actions (BC4) and enhance agency (WC4), which can further build self-efficacy.

#### Autonomy

Having a choice was one of the most important characteristics of volunteering expressed by respondents. Employees highlighted that they feel free to choose which voluntary action to engage in, and they described themselves as agents of decisions and actions. Managers and white-collar employees more often declared that they use a possibility to launch new projects (M5, WC5), while blue collar employees appreciated the opportunity to join others’ initiatives (BC5). The freedom also enabled employees to act in accordance with their values and interests (WC6).

Respondents stated that when it comes to volunteering, they do not feel controlled. There are no official schedules or implemented ways to monitor employee volunteering activities by the management (M6) and they can engage to a varying extent given the circumstances (BC6). Importantly, there are no rewards for voluntary work. Employees treat it as part of their job rather than something unusual.

### Discussion

In this case study, we showed that CSR activities can be construed so as to allow employees to create social bonds, to use strengths and build competence, as well as to be experienced as self-chosen and coherent with the self. Positive outcomes of helping for meaningfulness occur through the satisfaction of autonomy, competence and relatedness [18, 19]. MW, being an ‘abstract’ concept, may be hard to put into practice [19]. Our findings point to concrete organizational practices that are supportive of autonomy, competence, and relatedness, and give managers more executable suggestions on how to support employees’ MW.

The interviews uncovered that helping was perceived as an important aspect of employees’ identification to the company, allowing them to create shared organizational identity. This finding points to possible additional benefits of satisfying relatedness, competence, and autonomy needs in CSR actions. For instance, social identification has been linked with positive health outcomes [20] and lower turnover intentions [21]. Additionally, shared experiences and a possibility to discuss them allows employees to create ‘shared reality’, which increases interpersonal connectedness and trust [22, 23]. Purposeful recruitment which considers person-organization fit as it relates to congruence in values that relate to benevolence can facilitate this process [24].

While acting for the ‘greater good’ serves the communal MW fulfilment [8], respondents in our interviews pointed to additional personal benefits of voluntary work, such as competence development or increasing the meaning life. Thus, we contribute to the literature by showing that CSR activities may be instrumental in shaping both the agentic and communal MW. The design of the presented CSR initiative can serve the full MW identified by Lips-Wiersma [25] as a combination of developing and becoming self, expressing full potential, unity with others, and serving others.

To the best of our knowledge, this is the first case study which analyses the practical use of SDT in CSR solutions. Notably, SDT is a well-established framework and can provide evidence-based guidelines to design human resource management practices [11], including CSR solutions that support MW at work. Another strength of this case is its universality, since presented solutions are not industry-specific and can be adapted in different organizational contexts.

### Conclusions

Implementing SDT theory in organizational CSR programs can serve as an evidence-based method of enhancing social purpose in organization and employees’ MW. SDT initiatives should involve building connections, utilizing people’s strengths, as well as allowing for autonomy. By engaging workforces in volunteering that follows these principles, companies may not only build its positive image but also enable employees to experience their organization as positive, which is an important factor of well-being at work [26].

## Limitations

Some limitations need to be addressed. First, the interviews were drawn from one company in the private sector, which limits our ability to generalize the findings to the public sector [27]. Specifically, possibilities for empowerment may be limited in the latter context. Second, we only interviewed 15 employees; however, they represented distinct hierarchical levels (managers vs. non-managers) as well as position types (blue vs. white collar).

## Supplementary Information

**Additional file 1.** Interview guide for the study.

## Data Availability

The data used and/or analysed during the current study are available from the corresponding author on reasonable request.

## Abbreviations

MW: Meaning of work
CSR: Corporate social responsibility
SDT: Self-determination theory
