# Supplementary material for: Social purpose in an organization from the perspective of an employee: a self-determination outlook on the meaning of work
Source: BMC Res Notes. 2021 Jan 7;14:16. doi: 10.1186/s13104-020-05432-4 (PMC7791652; doi:10.1186/s13104-020-05432-4)
Supplement: Supplementary file 1 — Additional file 1. Interview guide for the study. [file 13104_2020_5432_MOESM1_ESM.docx]

Additional file 1. Interview guide

**At the beginning of the interview**

Hello, my name is Agnieszka Łądka-Barańska, and I am a PhD student at the University of Gdańsk. As part of a research project, I conduct interviews. Their theme is how employees perceive their work and the organisation in which they work.

Participation in this interview is voluntary, and you can resign from the further conversation at any time. You can also ask for information that has already been provided to be deleted.

The interview is anonymous, and the collected data will be presented in collective reports (i.e., on all persons in general, not individually). When presenting the results, we do not provide any information about your name or place of employment. We assign an identification number to all interviewees. The study was approved by the Department of Research Ethics of the University of Gdańsk.

To preserve the interview as faithfully as possible and to focus on our conversation, I would like to ask for the permission of recording this conversation with a voice recorder. The recording will be used only to prepare a recording of the conversation after the meeting, and until then, it will be stored only on a computer with password access. The recording files will be assigned to the ID numbers of the interviewees and deleted after completion of the survey. Documents with the consent to participate in the study are stored separately from the recordings, and it is not possible to combine the consent to participate with a given recording/statement.

**Are the terms of the interview understandable to you?**

▢Yes

▢No (if not - we explain doubts)

**Do you consent to the interview?**

▢Yes (if so - present consent to participate in the study for signing - Annex 1)

▢No (if not - end the interview)

**Questions**

| **Domain** | **Sample Questions** |
| --- | --- |
| **Job context** | Can you describe your role in the workplace?  How long have you been working here? |
| **Organizational context** | Do you know the values that your organization set as important? What are they?  What is the organizational vision and mission?  How are these values, goals, and mission/vision translated into practice? Please think about the different areas of the company's operation: work organization, management, communication with customers, communication with the employee. |
| **Volunteering program** | What are the charity activities in your company? Describe different areas and aspects that you are aware of.  To what extent do you participate in charity activities in your organization? What are you involved in?  Why are you taking part in charity activities in your organization?  To what extent do you have an impact on how to engage in them?  What consequences of the activities do you perceive for others? What are the consequences for yourself? |
| **Proactivity** | To what extent you have an impact on how to perform your work?  What actions did you take to change the way of performing your job?  What else do you have influence on in your work?  Have you been taking steps to develop your skills or learn new skills at work? If so, tell about them.  What new tasks, beyond the job description, have you undertaken?  Have you come up with any improvement in your work? What was it about?  What actions do you take to build positive relationships at work? |

**End of the interview**

- Thank the participant for the time spent on the study.
- Inform him/her that a collective feedback session will be organized at the end of the project, but if he/she wishes to obtain the results directly, he/she can ask to do so either now or by e-mail.
